# Restoration of Foveal Bulge after Resolution of Diabetic Macular Edema with Coexisting Serous Retinal Detachment

**DOI:** 10.1155/2020/9705786

**Published:** 2020-06-13

**Authors:** Yijun Hu, Qiaowei Wu, Baoyi Liu, Manqing Huang, Qingsheng Peng, Pingting Zhong, Xiaomin Zeng, Yu Xiao, Cong Li, Ying Fang, Tao Li, Honghua Yu, Xiaohong Yang

**Affiliations:** ^1^Guangdong Eye Institute, Department of Ophthalmology, Guangdong Provincial People's Hospital, Guangdong Academy of Medical Sciences/the Second School of Clinical Medicine, Southern Medical University, Guangzhou 510080, China; ^2^Aier Institute of Refractive Surgery, Refractive Surgery Center, Guangzhou Aier Eye Hospital, Guangzhou 510199, China; ^3^Aier School of Ophthalmology, Central South University, Changsha 410021, China; ^4^Shantou University Medical College, Shantou 515041, China; ^5^State Key Laboratory of Ophthalmology, Clinical Research Center for Ocular Disease, Zhongshan Ophthalmic Centre, Sun Yat-sen University, Guangzhou 510060, China

## Abstract

**Purpose:**

To evaluate the impact of restoration of foveal bulge (FB) in optical coherence tomography (OCT) images on visual acuity after resolution of diabetic macular edema with coexisting serous retinal detachment (SRD-DME).

**Methods:**

A total of 52 eyes with resolved SRD-DME and an intact ellipsoid zone at the central fovea were included. All eyes underwent best-corrected visual acuity (BCVA) examination and OCT scanning at baseline and follow-up visits (1, 3, and 6 months). The eyes were divided into two groups according to the presence of FB at 6 months. BCVA, central foveal thickness (CFT), height of SRD (SRDH), outer nuclear layer (ONL) thickness, photoreceptor inner segment (PIS), and outer segment (POS) length were compared between the two groups.

**Results:**

A FB was found in 25 of 52 (48%) eyes at 6 months. The FB (+) group had lower SRDH at baseline, and better BCVA, longer POS length at 6 months (all *P* < 0.05). There was no significant difference in the CFT, ONL thickness, and PIS length at 6 months between the two groups (all *P* > 0.05). More eyes in the FB (+) group had complete SRD resolution at 1 month (*P* = 0.009) and 3 months (*P* = 0.012). Eyes with complete SRD resolution at 1 month (*P* = 0.009) or 3 months (*P* = 0.012) were more likely to have a FB at 6 months.

**Conclusions:**

The Presence of the FB is associated with better BCVA after resolution of SRD-DME. Eyes with lower baseline SRDH or faster SRD resolution are more likely to have a FB at 6 months.

## 1. Introduction

Retinal detachment (RD) refers to a clinical situation where the neurosensory retina is detached from the underlying retinal pigment epithelium (RPE) [1]. Separation of the neurosensory retina from the RPE leads to deprivation of nutrition and oxygen supplies to the outer retina which in turn causes photoreceptor apoptosis and visual loss [1–3]. For instance, disruption of the ellipsoid zone (EZ), which represents the junction of the inner and outer photoreceptor segments, has been observed in patients with rhegmatogenous RD (RRD) using optical coherence tomography (OCT) [4, 5]. Successful reattachment of the neurosensory retina is essential for vision recovery in RD patients. Retinal reattachment allows restoration of blood supply to the outer retina and regeneration of the photoreceptors, and the patients' visual acuity is recovered accordingly. Better visual recovery is usually closely correlated to an intact EZ at the fovea after retinal reattachment [6, 7]. However, visual acuity in some RRD patients after successful retinal reattachment is still unsatisfactory despite the presence of an intact EZ at the fovea [8, 9]. In these patients, the absence of a foveal bulge (FB) is considered to be a reason for the incomplete visual recovery after retinal reattachment [8, 9].

Serous RD (SRD) is a subgroup of RD commonly seen in central serous chorioretinopathy (CSC) and diabetic macular edema (DME) [10, 11]. DME with coexisting SRD (SRD-DME) can be observed in the OCT images of DME patients [10]. Photoreceptor damage can also be seen in eyes with SRD-DME and the photoreceptors can be restored after resolution of the DME [12–14]. However, the visual acuity of some DME patients cannot be fully restored despite the complete edema resolution and presence of an intact EZ at the fovea. In light of the previous studies of RRD, we suppose the FB may have an impact on the visual acuity of these DME patients. In the present study, we aimed at determining whether the presence of a FB is correlated with a better visual acuity after the resolution of SRD-DME.

## 2. Methods

### 2.1. Subjects

In this retrospective study, 43 patients (52 eyes) with resolved SRD-DME were recruited from the Department of Ophthalmology at Guangdong Provincial People's Hospital (GPPH) between January 1, 2017, and March 1, 2019. All the patients received comprehensive baseline ophthalmologic examinations including best-corrected visual acuity (BCVA) with decimal chart which was converted to the logarithm of minimal angle of resolution (LogMAR) and the Snellen visual acuity, slit-lamp biomicroscope anterior segment and fundus examination, intraocular pressure (IOP) measurement, and baseline SD-OCT scanning (Spectralis; Heidelberg Engineering, Heidelberg, Germany). All patients underwent BCVA measurement, fundus examination, and SD-OCT scanning at 1, 3, and 6 months after 3 monthly consecutive intravitreal injections of ranibizumab (IVR) treatment. The study was conducted according to the 1964 Helsinki declaration and was approved by the Institutional Review Board of GPPH. Informed consent was obtained from all the patients after explanation of the nature of the study.

The inclusion criteria were SRD-DME secondary to type 2 diabetes mellitus (DM) and involving the fovea at baseline, SRD-DME resolution with an intact EZ at the central fovea at 6 months, treatment-naive eyes or eyes that received previous anti-VEGF or retinal photocoagulation no less than 6 months ago, BCVA between 0.3-1.0 LogMAR (≈20/200 - 20/40), and central foveal thickness (CFT) more than 275 *μ*m before treatment [15, 16]. We excluded eyes with macular edema or SRD secondary to other causes such as age-related macular degeneration, polypoidal choroidal vasculopathy, retinal artery/vein occlusion, CSC, rhegmatogenous retinal detachment, eyes with macular ischemia, glaucoma, IOP > 21 mmHg, severe cataracts, refractive error greater than 6 diopters (D), a history of vitrectomy or macular grid photocoagulation, or DME previously treated with intravitreal or periocular injection or retinal photocoagulation within 6 months. Eyes that could not be scanned using SD-OCT due to poor patient cooperation were also excluded.

### 2.2. Intravitreal Injection of Ranibizumab

All patients received 3 monthly consecutive 0.5 mg IVR. After the loading treatment, patients received an additional injection if they met any of the following criteria: (a) BCVA decrease of ≥0.1 LogMAR; (b) CFT increase of ≥100 *μ*m; or (c) BCVA decrease due to newly formed or enlargement of previous intraretinal cyst or SRD, as decided by the surgeons. Treatment was suspended if one of the following criteria were met: (a) stable vision over 3 consecutive visits, including the current visit evaluation, specifically no further BCVA improvement attributed to treatment at the 2 last consecutive visits; or (b) BCVA ≤ 0.0 LogMAR observed at the 2 last consecutive visits [17].

### 2.3. OCT Measurement and Classification of DME

**Figure 1:**
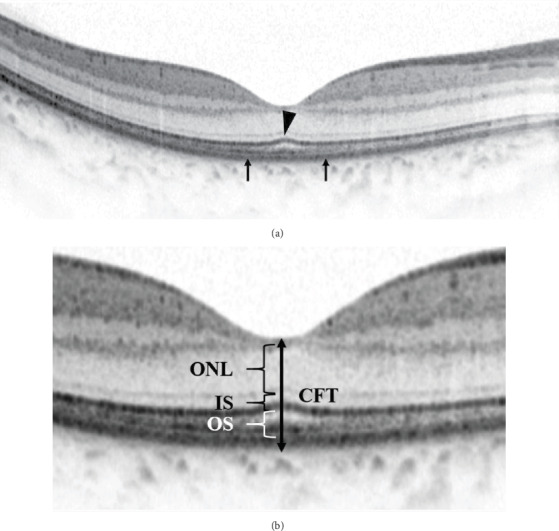
Illustration of spectral-domain optical coherence tomography (SD-OCT) image. (a) A horizontal 30° scan through the central fovea was obtained. The SD-OCT image shows that the photoreceptor inner segment/outer segment (PIS/POS) line has a bulge at the central fovea, named a foveal bulge (arrowhead). The foveal bulge is defined by the POS length at the central fovea being 10 *μ*m longer than the average POS length at 250 *μ*m temporal and nasal from the central fovea (arrows). (b) Magnified view. The CFT is the distance between the surface of the internal limiting membrane (ILM) and the outer border of the retinal pigment epithelium (RPE) at the central fovea. The thickness of the outer nuclear layer (ONL) is the distance between the outer border of the ILM and the outer border of the external limiting membrane (ELM). The length of the PIS is the distance between the outer border of the ELM and the outer border of the PIS/POS line. The length of the POS is the distance between the outer border of the PIS/POS line and the inner border of the RPE.

A custom 20°x 20° volume acquisition protocol was used to obtain a set of high-speed scans from each eye. With this protocol, 25 horizontal and central vertical cross-sectional B-scan images were obtained, each composed of 512 A-scans [18]. The horizontal image through the fovea as determined by simultaneous evaluation of the red-free image on the computer monitor of the OCT scanner [19] was exported for manual measurement of the CFT, height of SRD (SRDH), outer nuclear layer (ONL) thickness, photoreceptor inner segment (PIS), and outer segment (POS) length (Figure 1). OCT images were read and measured independently by 2 Chinese board-certified ophthalmologists (QW, BL) in a masked manner. If there were discordance between the 2 ophthalmologists, arbitration was performed by a retinal specialist (HY) to generate the final decision.

**Figure 2:**
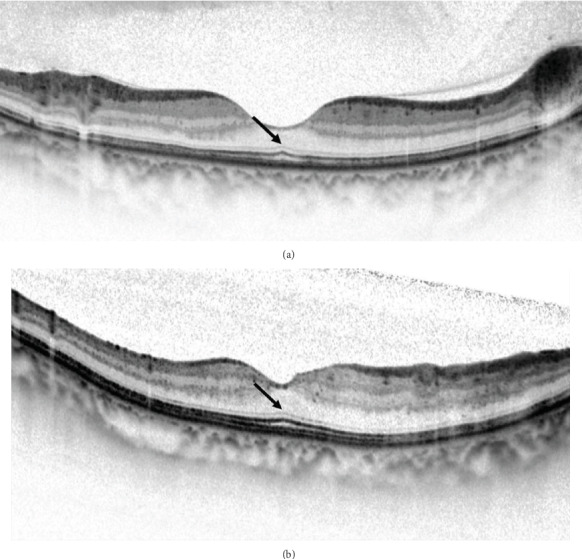
Spectral-domain optical coherence tomography (SD-OCT) images of eyes with diabetic macular edema. (a) The SD-OCT image of a 42-year-old woman 6 months after 3 monthly consecutive intravitreal injections of ranibizumab (IVR) treatment shows a complete resolution of DME with coexisting serous retinal detachment (SRD-DME). The SD-OCT image shows longer foveal photoreceptor outer segment (POS) length and the presence of a foveal bulge (arrow). The BCVA was 20/20. (b) The SD-OCT image of a 49-year-old man 6 months after 3 monthly consecutive IVR treatment shows a complete resolution of SRD-DME. The SD-OCT image also shows a longer foveal POS length and the presence of a foveal bulge (arrow). The BCVA was 20/25.

**Figure 3:**
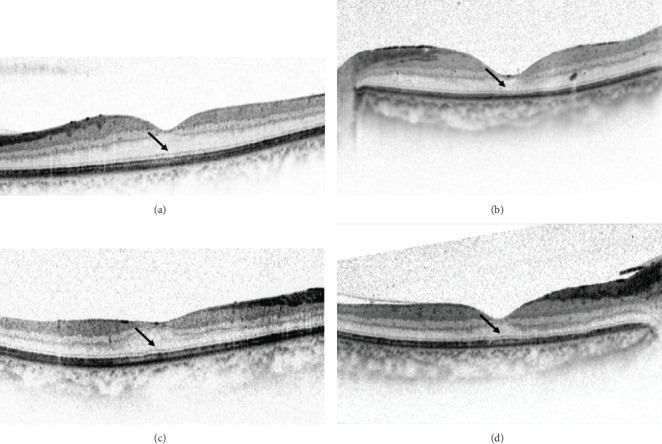
Spectral-domain optical coherence tomography (SD-OCT) images of resolved diabetic macular edema eyes without a foveal bulge (arrow). (a) The BCVA was 20/40. (b) The BCVA was 20/63. (c) The BCVA was 20/40. (d) The BCVA was 20/32.

SRD-DME was defined as DME with an elevation of the neurosensory retina and an optically clear space between the retina and RPE, with possible coexistence of intraretinal swelling or cysts in the macular area [17]. The eyes included were divided into two groups, the FB (+) group and the FB (-) group, based on the presence of FB at 6 months after IVR. The presence of the FB was defined as the POS length at the central fovea being 10 *μ*m longer than the average POS length at 250 *μ*m temporal and nasal from the central fovea [8, 9]. Typical OCT images of the FB (+) group and the FB (-) group are shown in Figures 2 and 3.

The CFT, SRDH, ONL thickness, PIS length, and POS length were manually measured at the central fovea. The CFT was defined as the distance between the surface of the internal limiting membrane (ILM) and the outer border of the RPE. The SRDH was defined as the vertical distance between the first signal from the top of the SRD and the signal from the anterior boundary of the RPE-choriocapillaris region. The ONL thickness was measured as the distance between the outer border of the ILM and the outer border of the external limiting membrane (ELM). The PIS length was the distance between the outer border of the ELM and the outer border of the PIS/POS line. The POS length was the distance between the outer border of the PIS/POS line and the inner border of the RPE [8, 9].

### 2.4. Statistical Analysis

The data are presented as mean ± standard deviation. All statistical analyses were performed using the SPSS 20.0 (SPSS. Inc., Chicago, IL, USA). To validate the agreement between the two ophthalmologists (QW, BL), the intraclass coefficient (ICC) was calculated. Statistical differences in the parameters between the FB (+) group and the FB (-) group were assessed using the unpaired Mann-Whitney test, Chi-square test, or Fisher's exact test after confirming the data normality. For all the tests, *P* < 0.05 was considered statistically significant.

## 3. Results

### 3.1. Basic Characteristics

A total of 52 eyes with completely resolved SRD-DME and an intact ellipsoid zone at the central fovea were included. A FB was found in 25 eyes (48%) at 6 months. Basic characteristics were not significantly different between the FB (+) group and the FB (-) group (Table 1). Regarding the reproducibility of OCT measurements, the interobserver ICC was 0.958 for ONL thickness, 0.847 of PIS length, and 0.910 for POS length, suggesting good reproducibility for OCT measurements between the two ophthalmologists (QW, BL).

### 3.2. BCVA and OCT Measurements at 6 Months

The FB (+) group had better BCVA and longer POS length at 6 months compared to the FB (-) group (Table 2). At 6 months, the BCVA was 0.19 ± 0.18 in the FB (+) group and 0.35 ± 0.18 in the FB (-) group (*P* = 0.004, unpaired Mann-Whitney test). There were 9 eyes with a BCVA ≥20/20, and 7 of the 9 had a FB and 2 of the 9 had no FB. In the FB (+) group, there were 7 of 25 eyes with a BCVA ≥20/20, compared to 2 of 27 eyes in the FB (-) group (*P* = 0.071, Chi-square test). The CFT at 6 months was 187.68 ± 27.00 *μ*m in the FB (+) group and 196.37 ± 29.54 *μ*m in the FB (-) group (*P* = 0.314). The ONL thickness at 6 months was 94.80 ± 15.53 *μ*m in the FB (+) group and 101.67 ± 18.17 *μ*m in the FB (-) group (*P* = 0.203). The POS length at 6 months was 41.48 ± 3.39 *μ*m in the FB (+) group and 31.44 ± 3.24 *μ*m in the FB (-) group (*P* < 0.001). The PIS length at 6 months was 31.28 ± 2.76 *μ*m in the FB (+) group and 30.70 ± 3.45 *μ*m in the FB (-) group (*P* = 0.289).

### 3.3. Factors Associated with FB Formation

Mean SRDH was 214.96 ± 85.01 *μ*m in the FB (+) group and 308.11 ± 186.27 *μ*m in the FB (-) group (*P* = 0.040). In the FB (+) group, there was 84.0% of the eyes having complete SRD resolution at 1 month, compared to 48.1% of the eyes in the FB (-) group (*P* = 0.009, Fisher's exact test). At 3 months, 96.0% of eyes in the FB (+) group had SRD resolution, compared to 66.7% of eyes in the FB (-) group (*P* = 0.012) (Table 2). On the other hand, 61.8% of the eyes with complete SRD resolution at 1 month had a FB at 6 months, and 22.2% of the eyes with residual subretinal fluid at 1 month had a FB at 6 months (*P* = 0.009). Moreover, 57.1% of eyes with complete SRD resolution at 3 months had a FB at 6 months, and 10.0% of eyes with residual subretinal fluid at 3 months had a FB at 6 months (*P* = 0.012) (Table 3).

## 4. Discussion

In the present study, a FB was found in 48.1% of eyes with resolved SRD-DME at 6 months after IVR. Eyes in the FB (+) group had faster SRD resolution at 1 and 3 months and better BCVA and longer POS length at 6 months compared to the FB (-) group. The results of our study were consistent with previous studies showing better BCVA and longer POS length in eyes with a FB after successful RRD repair or resolution of macular edema associated with branch retinal vein occlusion (BRVO) [8, 20]. Based on our best knowledge, there has been no previous study about FB formation in eyes with resolved SRD-DME. Therefore, our study may shed light to further investigations about prognostic factors of visual outcomes after DME treatment. In our study, the BCVA in eyes with resolved SRD-DME and an intact EZ at the fovea varied from 20/100-20/16, and 82.7% of the eyes had a BCVA<20/20. These findings suggest that an intact EZ may not be the only indicator of good visual recovery after SRD-DME resolution. According to our results, the presence of a FB and longer POS at the fovea were also possible indicators of better visual outcomes in eyes with an intact EZ after SRD-DME resolution.

Previous studies have shown that an intact EZ is associated with better visual recovery after RRD surgery [21–23]. However, visual acuity may be still unsatisfactory in some eyes despite an intact EZ after retinal reattachment [8, 24]. Hasegawa et al. proposed that the presence of a FB at the fovea was associated with better visual acuity after successful RRD repair. They observed a FB in all the eyes with macula-on RRD and only in 28.6% of eyes with macula-off RRD. BCVA was significantly better in eyes with a FB after successful RRD surgery. The authors proposed a mechanism of the FB formation in normal eyes which involved POS thinning, elongation, and density increase during the development of the fovea and the difference in width between the POS and PIS. They supposed that the absence of a FB after RRD repair was due to length shortening and density decrease of the POS [8].

SRD was present in 21.7%-38.5% of eyes with DME and was suggested to be caused by a breakdown of the outer blood-retinal barriers [17, 19, 25–27]. Photoreceptor damages such as POS shortening and EZ disruption have been observed in eyes with SRD-DME [17, 26]. It is very likely that the photoreceptors and EZ undergo a recovery process after SRD-DME resolution similar to the one after RRD repair. With elongation and increased density of the POS after SRD-DME resolution, a normal FB is formed at the fovea. Thus, the formation of a FB would be a sign of better anatomical recovery of the photoreceptors at the fovea after SRD-DME resolution. A better anatomical fovea in turn leads to more favorable visual outcomes. Therefore, the FB can be considered as an indicator of better anatomical recovery and a prognostic factor of better functional recovery in eyes with resolved SRD-DME. This theory could be verified by the results of our study. In our study, eyes in the FB (+) group had significantly longer POS length at 6 months after IVR than the FB (-) group, indicating better POS regeneration in the FB (+) eyes. Accordingly, the BCVA at 6 months in the FB (+) group was significantly better than the FB (-) group, suggesting more favorable visual outcomes in the FB (+) eyes. Since the mean CFT at 6 months were not significantly different between the two groups and an intact EZ was present in all of the eyes, it seemed that eyes in the FB (+) group had underwent better POS regeneration and elongation, leading to a FB formation and better visual acuity after SRD-DME resolution. It is noteworthy that the presence of a FB is associated with a higher likelihood of having better BCVA, but not a guarantee or requirement of having ≥20/20 vision. In the present study, 22.2% of eyes with a BCVA ≥20/20 did not have a FB and 72% of eyes with a FB had a BCVA<20/20.

In our study, we also aimed at finding out factors associated with the FB formation. We observed a lower mean baseline SRDH in the FB (+) group compared to the FB (-) group. It was possible that baseline photoreceptor damage was more severe in the FB (-) group. Previous studies have shown that photoreceptor damage is more severe in DME eyes with higher SRD [26]. With the increased distance between the photoreceptors and the RPE, deprivation of nutrition and oxygen supplies to the photoreceptors is more severe which may cause more photoreceptor damages. Moreover, the speed of subretinal fluid resolution after treatment may be faster in eyes with lower SRDH. A previous study has found that the speed of SRD resolution after the intravitreal injection of dexamethasone implant is negatively correlated with the baseline SRDH [28]. In our study, the proportions of eyes with complete SRD resolution at 1 month or 3 months were significantly higher in the FB (+) group than the FB (-) group. Basic research has also demonstrated that the photoreceptors begin to recover only after the retina is reattached [29, 30]. Taken together, eyes with a lower SRDH may experience faster photoreceptor regeneration to heal the less severe EZ damage in a shorter period after DME treatment.

In the present study, the POS length was significantly longer in the FB (+) group. However, the ONL thickness or PIS length was not significantly different between the two groups. Similar findings were also reported in previous studies about FB formation after successful RRD repair or resolution of SRD associated with BRVO [8, 20]. In Hasegawa et al.'s theory, POS elongation is critical for FB formation after RD reattachment [8, 20]. On the other hand, the ONL and PIS seem not associated with the FB formation. This is reasonable since the POS loss is one of the first and primary damages caused by RD [29–31]. These findings indicate the necessity of reducing POS damage and promoting POS recovery in the treatment of RD, including SRD-DME. Previously, two clinical studies demonstrated that postoperative POS length was correlated with postoperative BCVA in DME patients treated with vitrectomy [32] and in idiopathic epiretinal membrane (ERM) patients underwent vitrectomy surgery [33]. These findings are consistent with the result of our study showing better BCVA and longer POS length in the FB (+) group. Moreover, preoperative POS length was shown to be predictive of postoperative BCVA, indicating that preoperative POS length was a potential predictor of visual outcome after vitrectomy surgery in patients with DME or ERM [32, 33]. However, the preoperative POS length was difficult to obtain in our study due to the presence of SRD in the patients. Further investigation is needed to reveal the predictive value of preoperative POS length for postoperative BCVA after complete SRD-DME resolution.

There was a substantial number of eyes with a BCVA < 20/20 after SRD-DME resolution in our study, some of the eyes even with a FB. Similar findings were also observed in a previous study about resolved macular edema associated with BRVO [20]. This might be due to the delayed functional recovery of the retina after anatomical recovery in eyes with DME [34]. The results also indicate that there are other unknown prognostic factors associated with visual outcomes of DME that need to be further investigated. The proportion of eyes with a BCVA < 20/20 seemed to be higher in our study compared to Hasegawa et al. [20]. This was because all of the eyes in our study had SRD at baseline, compared to 67.7% of eyes in Hasegawa et al.'s study [20]. The presence of SRD at baseline has been known to affect the visual acuity after DME treatment [17, 19, 27].

Our study had some limitations. Firstly, the conclusions of our study could only be applied to SRD-DME eyes treated with IVR, rather than eyes treated with intravitreal injection of other anti-VEGF medications or steroids. Although similar findings may be observed after other DME treatments, the exact course of SRD resolution may vary among different treatments. Secondly, we did not investigate the molecular mechanism of the FB formation in eyes with resolved SRD-DME. Further prospective studies may reveal the molecular pathogenesis of the photoreceptor recovery and FB formation. Thirdly, poor DM control in some of the patients might be one of the reasons for the unsatisfactory visual outcomes after treatment in these patients. Moreover, further studies with a larger number of treatment-naïve eyes and longer follow-up period are needed to validate the results of our study.

In conclusion, we have found that the presence of the FB is associated with better BCVA after the resolution of SRD-DME. SRD-DME eyes with lower baseline SRDH or faster SRD resolution are more likely to have a FB at 6 months.

## Figures and Tables

**Table 1:** Baseline characteristics of diabetic macular edema eyes with coexisting serous retinal detachment in foveal bulge (+) group and foveal bulge (-) group.

	Foveal bulge (+) (*n* = 25)	Foveal bulge (−) (*n* = 27)	*P*
Mean age (SD) (years)	54.64 (8.83)	56.67 (13.50)	0.134^†^
Mean IOP (SD) (mmHg)	14.12 (2.19)	15.07 (2.73)	0.132^†^
Mean time since diagnosis of DM (SD) (years)	7.72 (3.71)	9.04 (5.42)	0.568^†^
Mean duration of DME before IVR (SD) (months)	7.99 (6.43)	10.50 (7.43)	0.161^†^
Mean HbA1C (SD) (%)	8.09 (1.30)	8.66 (2.60)	0.761^†^
Diabetic retinopathy severity (*n* (%))			0.746^‡^
NPDR	15 (60.0%)	15 (55.6%)	
PDR	10 (40.0%)	12 (44.4%)	
Photocoagulation treatment (*n* (%))	13 (52.0%)	14 (51.9%)	0.991^‡^
Mean baseline BCVA (SD) (logMAR/Snellen VA)	0.46 (0.24)/≈20/57.7	0.52 (0.15)/≈20/66.2	0.135^†^
Mean baseline CFT (SD) (*μ*m)	533.84 (178.69)	587.37 (235.75)	0.589^†^
Mean baseline SRDH (SD) (*μ*m)	214.96 (85.01)	308.11 (186.27)	0.040^†^

^†^Unpaired Mann-Whitney test; ^‡^Chi-square test. IOP: intraocular pressure; DM: diabetic mellitus; IVR: intravitreal injection of ranibizumab; HbA1c: glycosylated hemoglobin; NPDR: nonproliferative diabetic retinopathy; PDR: proliferative diabetic retinopathy; BCVA: best-corrected visual acuity; Snellen VA: Snellen visual acuity; CFT: central foveal thickness; SRDH: height of serous retinal detachment; SD standard deviation.

**Table 2:** Comparison of posttreatment optical coherence tomography measurements in diabetic macular edema eyes with coexisting serous retinal detachment in foveal bulge (+) group and foveal bulge (-) group.

	Foveal bulge (+) (*n* = 25)	Foveal bulge (-) (*n* = 27)	*P*
IVR (SD) (n)	4.00 (1.29)	4.19 (1.18)	0.460^†^
Mean 6M BCVA (SD) (LogMAR/Snellen VA)	0.19 (0.18)/≈20/31.0	0.35 (0.18)/≈20/44.8	0.004^†^
Mean 6M CFT (SD) (*μ*m)	187.68 (27.00)	196.37 (29.54)	0.314^†^
Mean ONL thickness (SD) (*μ*m)	94.80 (15.53)	101.67 (18.17)	0.203^†^
Mean photoreceptor IS length (SD) (*μ*m)	31.28 (2.76)	30.70 (3.45)	0.289^†^
Mean photoreceptor OS length (SD) (*μ*m)	41.48 (3.39)	31.44 (3.24)	<0.001^†^
SRD complete resolution (*n* (%))			
1 M	21 (84.0%)	13 (48.1%)	0.009^‡^
3 M	24 (96.0%)	18 (66.7%)	0.012^‡^

^†^Unpaired Mann-Whitney test; ^‡^Fisher's exact test. IVR: intravitreal injection of ranibizumab; 6 M: 6 months after 3 monthly consecutive intravitreal injections of ranibizumab treatment; BCVA: best-corrected visual acuity; Snellen VA: Snellen visual acuity; CFT: central foveal thickness; ONL: outer nuclear layer; IS: inner segment; OS: outer segment; SD: standard deviation; SRD: serous retinal detachment.

**Table 3:** Presence of a foveal bulge at 6 months in diabetic macular edema with coexisting serous retinal detachment eyes with complete serous retinal detachment resolution of at 1 or 3 months.

1 M	SRD complete resolution (+) (*n* = 34)	SRD complete resolution (-) (*n* = 18)	*P*
FB (+) (*n* (%))	21 (61.8%)	4 (22.2%)	0.009^†^

3 M	SRD complete resolution (+) (*n* = 42)	SRD complete resolution (-) (*n* = 10)	*P*
FB (+) (n (%))	24 (57.1%)	1 (10.0%)	0.012^†^

^†^Fisher's exact test. SRD: serous retinal detachment; 1 M: 1 month after 3 monthly consecutive intravitreal injections of ranibizumab treatment; 3 M: 3 months after 3 monthly consecutive intravitreal injections of ranibizumab treatment; FB: presence of foveal bulge at 6 months after 3 monthly consecutive intravitreal injections of ranibizumab treatment.

## Data Availability

The data used during the current study are available from the corresponding author on reasonable request.
